# scDAC: deep adaptive clustering of single-cell transcriptomic data with coupled autoencoder and Dirichlet process mixture model

**DOI:** 10.1093/bioinformatics/btae198

**Published:** 2024-04-11

**Authors:** Sijing An, Jinhui Shi, Runyan Liu, Yaowen Chen, Jing Wang, Shuofeng Hu, Xinyu Xia, Guohua Dong, Xiaochen Bo, Zhen He, Xiaomin Ying

**Affiliations:** Center for Computational Biology, Beijing Institute of Basic Medical Sciences, Beijing 100850, China; Center for Computational Biology, Beijing Institute of Basic Medical Sciences, Beijing 100850, China; Center for Computational Biology, Beijing Institute of Basic Medical Sciences, Beijing 100850, China; Center for Computational Biology, Beijing Institute of Basic Medical Sciences, Beijing 100850, China; Center for Computational Biology, Beijing Institute of Basic Medical Sciences, Beijing 100850, China; Center for Computational Biology, Beijing Institute of Basic Medical Sciences, Beijing 100850, China; Center for Computational Biology, Beijing Institute of Basic Medical Sciences, Beijing 100850, China; Center for Computational Biology, Beijing Institute of Basic Medical Sciences, Beijing 100850, China; Department of Bioinformatics, Institute of Health Service and Transfusion Medicine, Beijing 100850, China; Center for Computational Biology, Beijing Institute of Basic Medical Sciences, Beijing 100850, China; Center for Computational Biology, Beijing Institute of Basic Medical Sciences, Beijing 100850, China

## Abstract

**Motivation:**

Clustering analysis for single-cell RNA sequencing (scRNA-seq) data is an important step in revealing cellular heterogeneity. Many clustering methods have been proposed to discover heterogenous cell types from scRNA-seq data. However, adaptive clustering with accurate cluster number reflecting intrinsic biology nature from large-scale scRNA-seq data remains quite challenging.

**Results:**

Here, we propose a single-cell Deep Adaptive Clustering (scDAC) model by coupling the Autoencoder (AE) and the Dirichlet Process Mixture Model (DPMM). By jointly optimizing the model parameters of AE and DPMM, scDAC achieves adaptive clustering with accurate cluster numbers on scRNA-seq data. We verify the performance of scDAC on five subsampled datasets with different numbers of cell types and compare it with 15 widely used clustering methods across nine scRNA-seq datasets. Our results demonstrate that scDAC can adaptively find accurate numbers of cell types or subtypes and outperforms other methods. Moreover, the performance of scDAC is robust to hyperparameter changes.

**Availability and implementation:**

The scDAC is implemented in Python. The source code is available at https://github.com/labomics/scDAC.

## 1 Introduction

scRNA-seq is a technique for sequencing transcriptomes at single-cell resolution (Hwang *et al.* 2018). The analysis of scRNA-seq data helps to identify cell types based on gene expressions of different cells, which plays an important role in the studies of gene regulation mechanisms (Xu *et al.* 2016), organism development processes (Guo *et al.* 2019), etc. Single-cell clustering analysis is a vital step in identifying cell types and in revealing the heterogeneity and diversity of cells. It is also an essential process for downstream analysis.

There are many clustering methods that can be applied to scRNA-seq data (Duò *et al.* 2018). For instance, k-means performs clustering by iteratively updating K cluster centers (Hartigan and Wong 1979). SC3 obtains multiple groups of clustering results with different parameters in parallel and then performs hierarchical clustering to obtain final results (Kiselev *et al.* 2017). CIDR reduces dimensionality by principal coordinate analysis (PCoA) which takes the dropout events into account, and then performs hierarchical clustering (Lin *et al.* 2017). DBSCAN is a density-based spatial clustering nonparametric algorithm commonly used in many scenes (Ester *et al.* 1996). Louvain is a graph-based community detection clustering algorithm (Blondel *et al.* 2008); based on Louvain, Traag *et al.* (2019) proposed the Leiden algorithm, which made the clustering results more stable by adding a local optimization process. FFC is a graph-based method for clustering by iteratively propagating labels (Chen *et al.* 2022).

However, all the above algorithms have some nonnegligible limitations. One of the limitations is that in order to handle high dimensional scRNA-seq data, these algorithms decompose the clustering process into two separate steps: dimensionality reduction and clustering. Their performance relies on dimensionality reduction methods such as Principal component analysis (PCA) and Uniform manifold approximation and projection (UMAP) (Becht *et al.* 2019, McInnes *et al.* 2020). Since dimensionality reduction does not take subsequent clustering into consideration, they often lose information critical for clustering and thus tend to produce results that could not correspond well to biological meanings. Another limitation is that many algorithms are not applicable for large-scale scRNA-seq data, since the required memory usually scales quadratically with the number of cells. As scRNA-seq data increase rapidly, accurate and scalable clustering methods are urgently needed.

Some clustering methods based on deep learning have been presented to overcome the above limitations. Based on the AE, GLDC computes the clustering probability distribution of each sample in low-dimension representation by constructing a weighted adjacency matrix associated with a similarity graph of the samples (Huang *et al.* 2023). scDeepCluster is a deep clustering method based on the AE model and has a zero-inflated negative binomial (ZINB) layer to adapt to high dropout scenarios (Tian *et al.* 2019). But the strong assumption of ZINB makes scDeepCluster lack flexibility and may result in unsatisfactory clustering results (Svensson 2020). scGMAI and scVAE are the clustering methods using the Gaussian mixture model, which are based on AE and VAE architectures respectively (Grønbech *et al.* 2020, Yu *et al.* 2021). However, the clustering results of the Gaussian mixture model depend on the parameter of cell type number, and vary with the cell type number specified manually, which makes it difficult to achieve the optimal result. SDCN, scCCESS and scCAN employ classical clustering techniques after utilizing AEs to select relevant features (Bo *et al.* 2020, Tran *et al.* 2022, Yu *et al.* 2022). However, as the primary goal of AE is to reconstruct the original data, it may not inherently guarantee the extraction of features specifically optimized for clustering, potentially leading to unsatisfactory clustering results. scDHA combines two AE modules with k-nearest neighbor (kNN) (Tran *et al.* 2021). Since kNN tends to cluster cells into large cell groups, it often fails to detect rare cell types. The common advantage of these deep clustering methods is that they are all scalable to large volume of scRNA-seq data. However, they still suffer from limitations such as challenges in achieving optimal results and overlooking rare cell types. Moreover, all these methods cannot adaptively obtain the number of clusters. They require manual input of the cluster number by users or infer the cluster number by traversing multiple sets of parameters based on a certain measure.

As an important branch of deep learning, graph autoencoders (GAEs) have also been widely utilized in the clustering analysis of scRNA-seq data in recent years. Two notable examples are scGNN (Wang *et al.* 2021) and graph-sc (Ciortan and Defrance 2022), which are designed to capture structural relationships between cells and between cells and genes using GAEs. In addition, scMGCA (Yu *et al.* 2023) and CellVGAE (Buterez *et al.* 2022) are based on the architectures of graph convolutional autoencoders and variational graph autoencoders with graph attention layers, respectively. All of these methods employ classical clustering techniques on low-dimensional representations obtained through GAEs. However, the separation of the dimensionality reduction process and the clustering process often results in the loss of essential information during dimensionality reduction, potentially impairing clustering results. To overcome this disadvantage, several GAE methods that couple the dimensionality reduction process and clustering process have been proposed. One such method is scGAC (Cheng and Ma 2022), which is based on the graph attention autoencoder. By employing an iterative self-optimizing clustering method, scGAC allows the dimensionality reduction and clustering modules to mutually benefit each other, thereby enhancing clustering results. However, scGAC requires users to specify the number of cell types.

In fact, it is quite important to adapt cluster numbers for clustering method to discover *bona fide* heterogeneity in scRNA-seq data. In many application scenarios, it is impossible to know the number of clusters in advance. Arbitrarily specifying cluster numbers leads to misguided clustering results and hence results in biological discoveries prone to error. For those clustering methods that obtain cluster numbers by traversing parameters, workload and time cost are nonnegligible issues, especially in large scale datasets. Also, these methods require users to have experience in judging whether the parameters are optimal.

Here, we propose a deep adaptive clustering method scDAC based on coupled AE (Hinton and Salakhutdinov 2006) and DPMM (Antoniak 1974). scDAC takes advantage of the AE module to be scalable, and takes advantage of the DPMM module to cluster adaptively without ignoring rare cell types. To achieve accurate clustering, we couple AE and DPMM by jointly optimizing the parameters of the two modules, so that the dimensionality reduction by AE is constrained by DPMM clustering. We then evaluate the performance of scDAC on five subsampled datasets and nine scRNA-seq datasets. The results show that scDAC can cluster scRNA-seq data adaptively and accurately. Moreover, scDAC is robust to changes in hyperparameters.

## 2 Materials and methods

### 2.1 The architecture of scDAC

**Figure 1.**
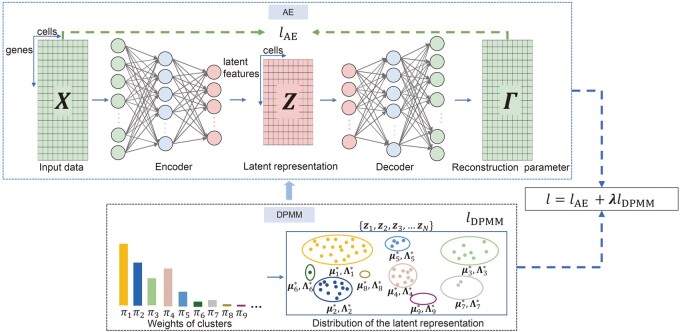
The overview of the scDAC framework. Upper panel: we obtain the low-dimensional representation $Z=[z_{1};z_{2};\cdots ;z_{N}]\in R^{N\times D}$ of input data $X=[x_{1};x_{2};\cdots ;x_{N}]\in N^{N\times G}$ through scDAC’s AE module. $\Gamma =[\eta_{1};\eta_{2};\cdots ;\eta_{N}]\in R_{+}{\;}^{N\times G}$ is the parameter matrix for input reconstruction. Lower panel: the low-dimensional representation $z_{n}$ obeys the DPMM, and is assumed to be generated from a Gaussian with mean $\mu_{y_{n}}^{*}\in \{\mu_{1}^{*},\mu_{2}^{*},\ldots \}$ and precision matrix $\Lambda_{y_{n}}^{*}\in \{\Lambda_{1}^{*},\Lambda_{2}^{*},\ldots \}$, where $y_{n}$ is the cluster label of $z_{n}$ and obeys the Categorical distribution parameterized by cluster weights $\left[\pi_{1},\pi_{2},\ldots \right]$. Different labels are indicated by different colors in the lower panel.

We propose scDAC, a deep unsupervised adaptive clustering model for scRNA-seq data. The input data for scDAC are the expression matrix of *N* cells and *G* genes. The output is the cluster label vector of *N* cells. scDAC is based on the AE structure. For cell *n*, the gene expression vector $x_{n}\in N^{G}$ is fed into the AE module to obtain the nonlinear dimensionality reduction result $z_{n}\in R^{D}$. Meanwhile, a constraint is given to $z_{n}$ through the prior distribution from the DPMM module. We define the loss as the AE’s reconstruction error of $x_{n}$ and the negative log joint probability of $z_{n}$ and DPMM latent variables. During training, the loss is minimized by alternatively optimizing the parameters of AE and DPMM. After training, we output the cluster label $y_{n}\in N_{+}$ of each cell, which is inferred from $z_{n}$ through the DPMM module. The workflow of scDAC is shown in Fig. 1.

### 2.2 Autoencoder module

We use AE to achieve nonlinear dimensionality reduction of the input data $x_{n}$ and maintain the biological information. It is defined as follows:

(1) $$z_{n}=f(x_{n};\theta^{f})$$

 

(2) $$\begin{array}{c} \eta_{n}=g(z_{n};\theta^{g}) \end{array}$$

where the encoder network *f*, with parameter $\theta^{f}$, converts the input $x_{n}$ into a low-dimensional representation $z_{n}$. The decoder network *g*, with parameter $\theta^{g}$, predicts the distribution of $x_{n}$ conditioned on $z_{n}$, which is assumed to be a Poisson parameterized by $\eta_{n}$. We define the AE’s reconstruction loss $l_{\text{AE}}$ as the negative log-likelihood of $\eta_{n}$ given $x_{n}$, i.e.:

(3) $$\begin{array}{c} \begin{array}{c} -\ln p(x_{n}|\eta_{n})=-\ln\text{Poisson}(x_{n}|\eta_{n})\\ =-\ln\text{Poisson}(x_{n}|g(f(x_{n};\theta^{f});\theta^{g}))\\ ≜l_{\text{AE}}(\theta ;x_{n}) \end{array} \end{array}$$

where $\theta =\{\theta^{f},\theta^{g}\}$ is the set of encoder and decoder parameters.

### 2.3 The module of Dirichlet process mixture model

To achieve adaptive clustering, we impose a DPMM prior on the low-dimensional representation so that the cluster number can be inferred automatically. We assume $z_{n}$ is generated from a DPMM, which is defined as follows:

(4) $$p(\delta_{k})=\text{Beta}(\delta_{k}|1,\alpha ),k=1,2,\ldots$$

 

(5) $$\begin{array}{c} \pi_{k}=\delta_{k}\prod_{j=1}^{k-1}\left(1-\delta_{j}\right),k=1,2,\ldots \end{array}$$

 

(6) $$\begin{array}{c} \pi =[\pi_{1},\pi_{2},\ldots ] \end{array}$$

 

(7) $$\begin{array}{c} p(y_{n}|\pi )=\text{Categorical}(y_{n}|\pi ) \end{array}$$

 

(8) $$\begin{array}{c} p(\mu_{k}^{*},\Lambda_{k}^{*})=\text{NW}(\mu_{k}^{*},\Lambda_{k}^{*}|\sigma ,1,D,\zeta ),k=1,2,\ldots \end{array}$$

 

(9) $$\begin{array}{c} p(z_{n}|\mu_{y_{n}}^{*},\Lambda_{y_{n}}^{*})=\text{Normal}(z_{n}|\mu_{y_{n}}^{*},\Lambda_{y_{n}}^{*}{\;}^{-1}) \end{array}$$

where the variable $\delta_{k}\in (0,1)$ obeys the Beta distribution parameterized by 1 and α, and α > 0 is a hyperparameter set empirically. $\pi_{k}$ is the probability of a cell belonging to the *k*th cluster, satisfying $\pi_{k}\in (0,1)$ and $\pi_{1}\;+\;\pi_{2}\;+\;\cdots =1$. $y_{n}$ is the cluster label obeying the Categorical distribution parameterized by π. $\mu_{k}^{*}$ and $\Lambda_{k}^{*}$ are sampled from the Normal–Wishart (NW) distribution parameterized by σ, 1, *D*, and ζ, where σ and ζ are the hyperparameters empirically set as the mean and the variance of the low-dimensional representations of all cells, respectively. Given the cluster label $y_{n}$, $z_{n}$ is generated from a Gaussian with mean $\mu_{y_{n}}^{*}$ and precision matrix $\Lambda_{y_{n}}^{*}$.

Let $S=\{y_{n},\delta_{1},\delta_{2},\ldots ,\mu_{1}^{*},\mu_{2}^{*},\ldots ,\Lambda_{1}^{*},\Lambda_{2}^{*},\ldots \}$ be the set of all DPMM variables. Our aim in this module is to maximize the joint probability p(S,Z), and here we adopt an iterative learning approach. Concretely, we first assume Z is known and minimizing the term [−lnp(S,Z)], which can be rewrite as:

(10) $$\begin{array}{c} \begin{array}{c} -\ln p(S,Z)\\ =-\ln p(Z)-\ln p(S|Z)\\ =\;\text{const}.-\ln p(S|Z) \end{array} \end{array}$$

For [−lnp(S|Z)], we further have:

(11) $$\begin{array}{c} \begin{array}{c} -\ln p(S|Z)\\ =-\ln p(S|z_{1},z_{2},\ldots ,z_{N})\\ =-\ln p(S|f(x_{1};\theta^{f}),\ldots ,f(x_{N};\theta^{f}))\\ ≜\;l_{\text{DPMM}}^{S}(S;X,\theta^{f}) \end{array} \end{array}$$

In this case, the minimization of [−lnp(S,Z)] can be achieved by minimizing the loss term $l_{\text{DPMM}}^{S}$ with respect to S.

Then, assuming S is known, we can achieve the minimization of the term [−lnp(S,Z)] as follows:

(12) $$\begin{array}{c} \begin{array}{c} -\ln p(S,Z)\\ =-\ln p(S)-\ln p(Z|S)\\ =\;\text{const}.+\sum_{n}(-\ln p(z_{n}|S)) \end{array} \end{array}$$

For $\left[-\ln p(z_{n}|S)\right]$, we further have:

(13) $$\begin{array}{c} \begin{array}{c} -\ln p(z_{n}|S)\\ =-\ln p(f(x_{n};\theta^{f})|S)\\ ≜\;l_{\text{DPMM}}^{z}(\theta^{f};x_{n},S) \end{array} \end{array}$$

Thus, the minimization of [−lnp(S,Z)] is equivalent to minimizing the loss term $l_{\text{DPMM}}^{z}$ with respect to $\theta^{f}$ for all cells.

### 2.4 Loss function

In order to achieve dimensionality reduction and adaptive clustering simultaneously, we define the loss function for updating the parameters of encoders and decoders in the scDAC, which combines the losses defined in Equations (3) and (13) for both modules, respectively, i.e.:

(14) $$\begin{array}{c} l(\theta ;S,x_{n})≜l_{\text{AE}}(\theta ;x_{n})+\lambda l_{\text{DPMM}}^{z}(\theta^{f};x_{n},S) \end{array}$$

where λ>0 is the hyperparameter.

### 2.5 Training and inference

The minimization of the loss $l(\theta ;S,x_{n})$ in Equation (14) requires the value of S. However, S is unknown and the learning of S depends on $\theta^{f}\in \theta$ as shown in Equation (11). Here we adopt an iterative approach to minimize Equations (11) and (14), obtaining training algorithm of scDAC (Algorithm 1). In Algorithm 1, *T* is the total number of iterations, and *I* is the number of iterations in each epoch. For inference, the cluster label $y_{n}$ is obtained through the maximum a posteriori estimation, i.e. we assign $y_{n}=\underset{k}{\arg\max}(\pi_{nk})$ with the index corresponding the maximum value in $\pi_{n}$.

Algorithm 1. The scDAC training algorithm.

**Input**: A single-cell RNA-seq dataset ${\left\{x_{n}\right\}}_{n\in N}$

**Output**: The set parameters θ in the AE module and the set of latent variables S in the DPMM module

1: **for**  t=1,2,…,T  **do**

2:  Sample a mini-batch ${\left\{x_{n}\right\}}_{n\in N_{t}}$ from the dataset, where $N_{t}\subset N$

3:  Freeze S and update θ via SGD with loss $\frac{1}{\left|N_{t}\right|}\sum_{n\in N_{t}}l(\theta ;S,x_{n})$  ▹ See Equation (14)

4:  **if**  t mod I=0  **then**

5:   Freeze θ and update S via SGD with loss $\frac{1}{\left|N_{t}\right|}\sum_{n\in N_{t}}l_{\text{DPMM}}^{S}(S;X,\theta^{f})$  ▹ See Equation (11)

6:  **end if**

7: **end for**

### 2.6 Datasets

In this study, we use 10 publicly available scRNA-seq datasets from different sequencing platforms, species, organs, experimental conditions, and scales. Details are shown in Supplementary Table S1.

In order to verify the adaptive clustering ability and robustness to the parameter of scDAC, we subsample from the Chen dataset (Chen *et al.* 2017) and construct five subsampled datasets. The details are as follows: we select 10 cell types and sort them alphabetically by type names (the selected types are shown in Supplementary Table S2). We obtain five subsampled datasets by choosing the top 2, 4, 6, 8, and 10 types respectively, which are used in verifying the adaptive clustering ability. The subsampled dataset with 10 cell types is used for evaluating the robustness to changes in hyperparameters.

The datasets of Baron (Baron *et al.* 2016), Bhattacherjee (Bhattacherjee *et al.* 2019), Ghanem (Ghanem *et al.* 2022), Kozareva (Kozareva *et al.* 2021), Muraro (Muraro *et al.* 2016), Orozco (Orozco *et al.* 2020), Segerstolpe (Segerstolpe *et al.* 2016), Syper and Zilionis (Zilionis *et al.* 2019) are used to evaluate the performance of scDAC and the other 15 widely used clustering methods.

### 2.7 Experimental setting

UMI count matrices are normalized and log-transformed using the NormalizeData function in Seurat package (Hao *et al.* 2021) (v.4.1.0). We obtain the gene expression matrix after selecting 4000 highly variable genes using the FindVariableFeatures function and scaling the selected genes using the ScaleData function. The expression matrix is used as the input of scDAC and other methods which require raw data.

We implement the scDAC model using PyTorch (Paszke *et al.* 2019) (v.1.12.0) in Python 3 (v.3.7.11). For the DPMM module, we use the BayesianGaussianMixture function from scikit-learn (v.1.0.2).

The parameter details of scDAC are as follows: in the AE module, we set the sizes of two hidden layers of encoder to 256 and 128, set the sizes of the hidden layers of decoder to 128 and 256, and set the dimensionality of the low-dimensional representation to 32; in the DPMM module, the hyperparameter α is set to 1e−10 in all experiments except the robustness verification experiment; in the loss function, we set the hyperparameter λ to 1 in all experiments. During the training process, the minibatch size is set to 512, the AdamW (Loshchilov and Hutter 2019) optimizer with a fixed learning rate of 1e−4 is used for optimization.

During the training process, we first pre-train the AE module until a stable low-dimensional representation is obtained. Then we train the whole model including the AE and the DPMM modules.

### 2.8 Evaluation metrics

To evaluate the clustering performance of scDAC and other methods, we use four metrics, i.e. three classical clustering performance indicators and one indicator defined by us. The three classical indicators are Normalized Mutual Information (NMI) (Strehl *et al.* 2002), Adjusted Rand Coefficient (ARI) (Hubert and Arabie 1985) and Silhouette Coefficient (SC) (Rousseeuw 1987), which are implemented using scikit-learn (v.1.0.2). In order to evaluate cluster number adaptively, we put forward an indicator named Deviation Ratio. These metrics are detailed as follows.

NMI. NMI measures the degree of association between predicted labels and the ground truth, and it is the normalized result of mutual information (MI) score. The higher of the associative degree, the better of the performance of clustering method on the dataset. NMI score ranges from 0 to 1, where 0 and 1 correspond to no mutual information and perfect correlation, respectively.

ARI. Similar to NMI, ARI is used for evaluating the similarity between the predicted labels and the true ones. As a statistical method, ARI is the scaling of the Rand Coefficient (RI) score. ARI score ranges from 0 to 1, where 0 and 1 correspond to the random match and the exact match between the predicted labels and the ground truth.

SC. SC is an indicator describing the separation of clusters annotated by the predicted labels, and it compares the gap between the maximum distance of within-cluster and the minimum distance of between-cluster. Here, we use SC to evaluate the plausibility of predicted labels annotated on the low-dimensional representations. The value of SC is between −1 and 1, in order to calculate the average score of NMI, ARI, and SC, the value is scaled to 0–1. The value 0 corresponds to the worst situation that the points with different labels are clustered together; the value 1 corresponds to the best situation that the points with different label are clustered separately.

Deviation Ratio. Deviation Ratio (DR) is an indicator of deviation between the number of predicted labels and the number of true one, namely:

(15) $$\text{DR}=\frac{k_{\text{predicted}}-k_{\text{true}}}{k_{\text{true}}}$$

where $k_{\text{true}}$ is the number of true cell types and $k_{\text{predicted}}$ is the number of predicted clusters. A negative value of DR implies that the number of predicted labels is smaller than the number of real one, and vice versa. The larger absolute value of DR means the greater deviation, and 0 indicates that the number of predicted clusters equals to the number of true labels.

### 2.9 Comparing methods

We compared scDAC with commonly used clustering methods, including AE+DP, AE+k-means, PCA+k-means, AE+DBSCAN, PCA+DBSCAN, CIDR, SC3, scCCESS, AE+Louvain, PCA+Louvain, AE+Leiden, PCA+Leiden, GLDC, SDCN, and UMAP+FFC.

AE+DP. AE+DP uses the DPMM to cluster cells into different groups after obtaining the low-dimensional representation of data through AE. Dimensionality reduction and clustering are two separate steps. The implementation details of AE+DP are as follows: all of the parameter settings are the same as scDAC mentioned above, except for the hyperparameter α of DPMM. The hyperparameter α traverses 1e − 20,1e − 10,1e − 5,1e − 2,1e + 0,1e + 2, and 1e + 5. We select the clustering result with the highest ARI value among the traversal results as the final one. The predicted clusters are truncated since the cluster numbers are usually extremely large. We sort the clusters by size and maintain the dominant clusters that contain 95% of the cells in total. The remaining small clusters are uniformly labeled as NA. In fact, AE+DP is a simplified situation of scDAC without the joint optimization of AE and DPMM.

k-means. k-means clustering is often used after dimensionality reduction on scRNA-seq data. PCA or AE reduces the dimensionality of expression matrix to 32. The hyperparameter of cluster numbers of k-means is set to the number of true labels in each experiment. The k-means is implemented with the KMeans function in scikit-learn (v.1.0.2).

DBSCAN. DBSCAN clustering is often used after dimensionality reduction on scRNA-seq data. We use the same PCA or AE results as those in k-means. The hyperparameters of DBSCAN are eps (the maximum distance between two samples) and min samples (the number of samples or total weight in a neighborhood), which traverse 1–10 and 1–100, respectively. We select the clustering result with the highest ARI value among the traversal results as the final one. The DBSCAN is implemented with the DBSCAN function in scikit-learn (v.1.0.2).

CIDR, SC3, scCCESS, GLDC and SDCN. CIDR, SC3, scCCESS, GLDC, and SDCN are implemented with the original code. The hyperparameters of dimensionality reduction are set to 32. The hyperparameter of cluster numbers of GLDC and SDCN are set to the number of true labels in each experiment, and the other hyperparameters are set to default in our experiments.

Louvain and Leiden. Louvain and Leiden are often used after dimensionality reduction on scRNA-seq data. We use the same PCA or AE results as those in k-means. Both methods need to calculate the neighborhood before clustering. The parameters are set as follows: the hyperparameters of the calculation process of neighborhood include the number of neighbors and the number of components, which traverse 15, 30, 50, 100 and 5, 10, 15, 20, 25, 30, 32, respectively; the resolution parameter of Louvain and Leiden traverses 0.01, 0.1, 1, 10, and 100. We select the clustering result with the highest ARI value among the traversal results as the final one. Louvain/Leiden is implemented using the Neighbors and Louvain/Leiden function in scanpy (v.1.8.1).

FFC. FFC clustering is performed with UMAP results as inputs, since the authors claimed that UMAP dimensionality reduction leads to the best results than other methods. We set the dimensionality of the low-dimensional representation to 32. The implementation details of FFC are as follows: we set the two hyperparameters, neighbor number and fire temperature, to traverse 15, 30, 50, 100 and 10, 24, 50, 100, 150, 200, 250, 300, 350, 400, respectively. We select the clustering result with the highest ARI value among the traversal results as the final one.

## 3 Results

### 3.1 scDAC enables adaptive clustering of scRNA-seq data and is robust to hyperparameter changes

To verify the adaptive clustering performance and robustness to parameters changes of scDAC, we conducted two groups of experiments on the subsampled datasets. In the first group of experiments, we performed clustering on the five subsampled datasets containing 2, 4, 6, 8, and 10 cell types from Chen dataset. In the other group of experiments, we conducted clustering on the subsampled dataset with 10 cell types, setting the hyperparameter α to 10 different values in the wide range of [1e−50,1e+20].

**Figure 2.**
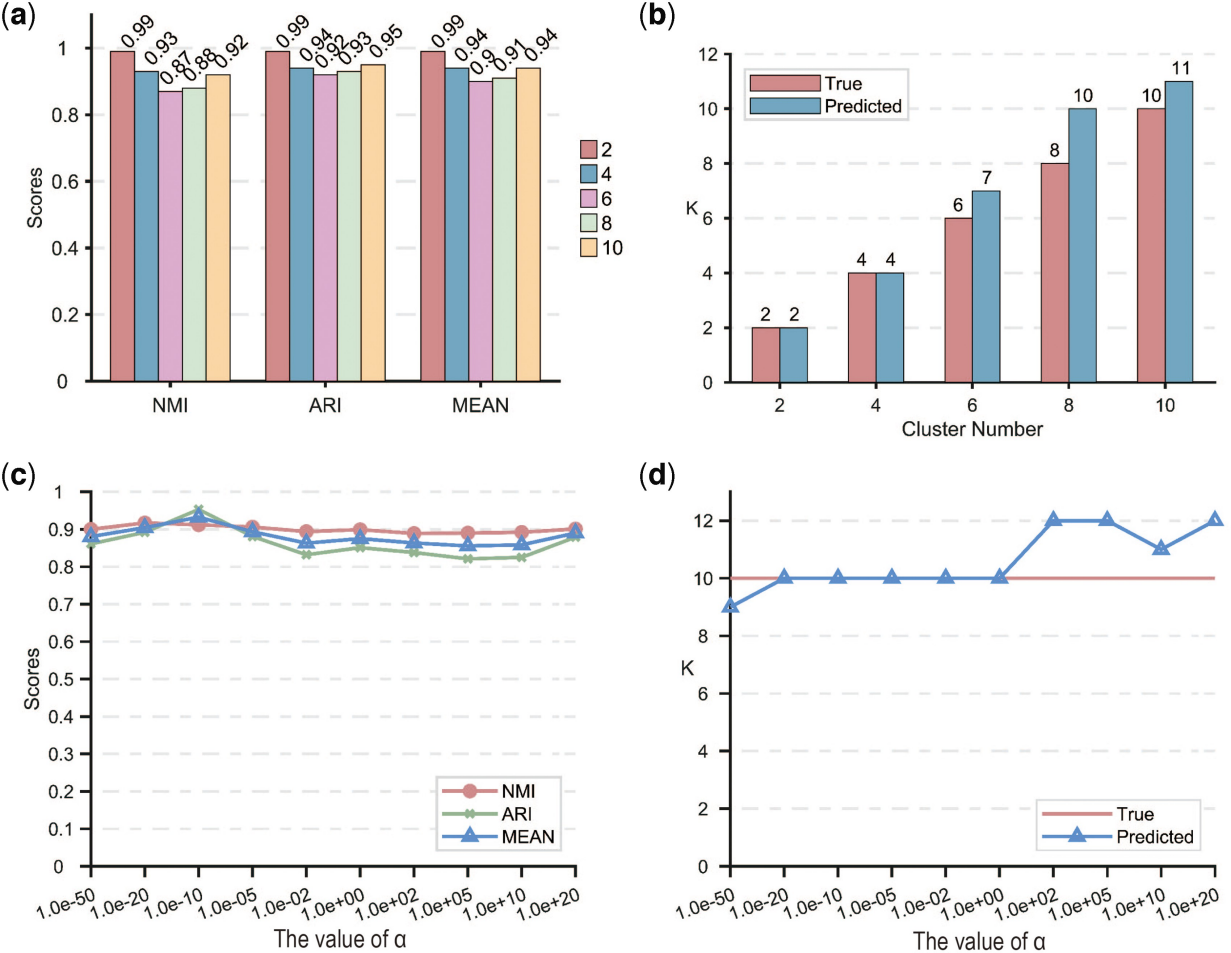
The performance of adaptivity and robustness of scDAC on subsampled datasets. (a) NMI, ARI, and their average scores on the five subsampled datasets with 2, 4, 6, 8, and 10 cell types. Higher bars represent better performance. (b) The comparisons between the numbers of clusters obtained by scDAC and those of true labels. The left bar represents the number of true labels, and the right bar represents the number of predicted clusters. (c) NMI, ARI, and their average scores under wide-range values of hyperparameter α. The *x* axis represents the values of α, the y axis represents the corresponding scores, and the different lines represent different kinds of indicators. (d) The comparison between the cluster numbers obtained by scDAC and those of true labels under different values of α. The *x* axis represents the values of α, and the *y* axis represents the numbers of labels, where the straight line represents the number of true labels, and the line with triangles represents the number of predicted labels.

In order to demonstrate the clustering performance of scDAC, we obtained the predicted labels on the five subsampled datasets and calculated the metrics of NMI, ARI and their average to reflect the similarity between the true labels and the predicted labels. The metrics are >0.85 in all the experiments (Fig. 2a), suggesting that the predicted labels are highly similar to the true labels. These results demonstrate that the clustering results of scDAC are accurate.

To verify the adaptive ability of scDAC, we compared the numbers of predicted clusters in the above experiments with those of true cell types. As shown in Fig. 2b, the numbers of clusters obtained by scDAC are very close to the numbers of the true one, suggesting that scDAC can adaptively obtain accurate cluster numbers and the performance is stable on the datasets with different numbers of cell types.

In order to evaluate the robustness to parameter changes of scDAC, we performed clustering with different values of the hyperparameter α on the subsampled dataset with 10 cell types. The hyperparameter α was set to 10 values in wide range: 1e − 50,1e − 20,1e − 10,1e − 5,1e − 2,1e + 0,1e + 2,1e + 5,1e + 10, and 1e + 20. NMI, ARI and their average were computed. The indicators are all >0.8 (Fig. 2c), suggesting high similarity between the predicted labels and the true one. We also compared the numbers of predicted clusters with that of true cell types. As shown in Fig. 2d, the number of predicted clusters increased slowly as parameter α increased, which is consistent with the meaning of α in the Dirichlet process model: the larger α leads to the more uniform distribution of cluster weights and hence leads to the greater number of clusters. Nonetheless, the numbers of predicted labels are all close to that of true labels, and the deviations are no >2, suggesting that scDAC can obtain accurate numbers of clusters despite the wide variation of the hyperparameter. These results demonstrate that scDAC is robust to changes in the hyperparameter.

### 3.2 scDAC shows superior clustering performance on various scRNA-seq datasets

**Figure 3.**
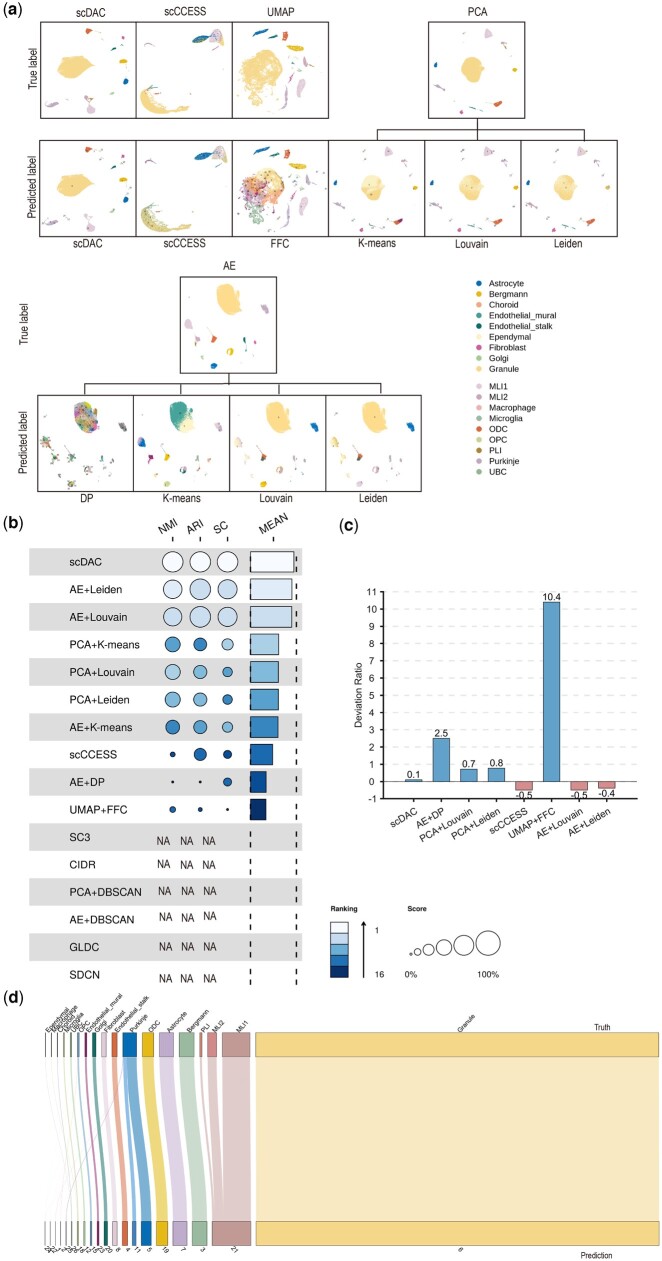
Comparisons of clustering performance between scDAC and the other 15 widely used methods on the Kozareva dataset. (a) UMAP visualization of the low-dimensional representations of these methods. The upper panel is annotated with true labels and the lower panel is annotated with predicted labels. The first column is the UMAP plot of scDAC, and the other columns are the plots of other methods. (b) NMI, ARI, SC, and mean scores of scDAC and the other methods. Each row represents a clustering method. The three columns of circles from left to right represent clustering indicators NMI, ARI, and SC scores, respectively. The rectangles on the right represent the mean scores of these three indicators. The size of the circles and rectangles correspond to the scores: the bigger one means the better performance. The darkness of color of the circles and rectangles correspond to the ranking: the lightest one means the top rank. The clustering methods are sorted according to the mean scores in descending order. NA denotes that the method failed to produce clustering results on this dataset. (c) The bar plot of Deviation Ratios between the predicted labels by different methods and the true one. The *x* axis represents different methods, and the *y* axis represents the Deviation Ratio value. Shorter bars represent better results. AE+k-means, PCA+k-means, GLDC, and SDCN were not involved in DR comparison since they require input of cell type number. (d) The Sankey plot of scDAC shows the correspondence between the predicted labels and the ground truth. SC3, CIDR, PCA+DBSCAN, AE+DBSCAN, GLDC, and SDCN crashed and failed to produce results on this dataset. Therefore, the corresponding results are not depicted or marked as NA in the figure.

We performed clustering experiments with scDAC and 15 widely used methods on Baron, Bhattacherjee, Ghanem, Kozareva, Muraro, Orozco, Segerstolpe, Syper, and Zilionis datasets from different sequencing platforms, species, organs, experimental conditions, and scales. The low-dimensional representations of these methods were visualized by UMAP with true labels and predicted labels annotated. The results show that the predicted labels by scDAC matched quite well with the true labels, surpassing the other clustering methods (Fig. 3a, panel a in Supplementary Figs S1–S7). The NMI, ARI, SC, and their mean scores are shown in Fig. 3b, panel b in Supplementary Figs S1–S7 and Supplementary Tables S3–S6. scDAC has the top three or top four high NMI, ARI, and SC scores among all the methods on all eight datasets. The mean scores combining all three indicators of scDAC is the highest among all the methods on all the datasets, demonstrating its superior clustering performance to the other methods. We also computed DRs that reflect the accuracy of the number of predicted labels by scDAC and the other 15 clustering methods. scDAC has the lowest DRs on all eight datasets (Fig. 3c, panel c in Supplementary Figs S1–S7), suggesting that scDAC can achieve more accurate cluster numbers than the other methods. We further investigated the relationship between the predicted labels of scDAC and the ground truth. Sankey plots show that the predicted labels by scDAC match the ground truth well and rare types are also identified accurately (Fig. 3d, panel d in Supplementary Figs S1–S7).

### 3.3 scDAC adaptively identifies fine-grained clusters on the scRNA-seq dataset

**Figure 4.**
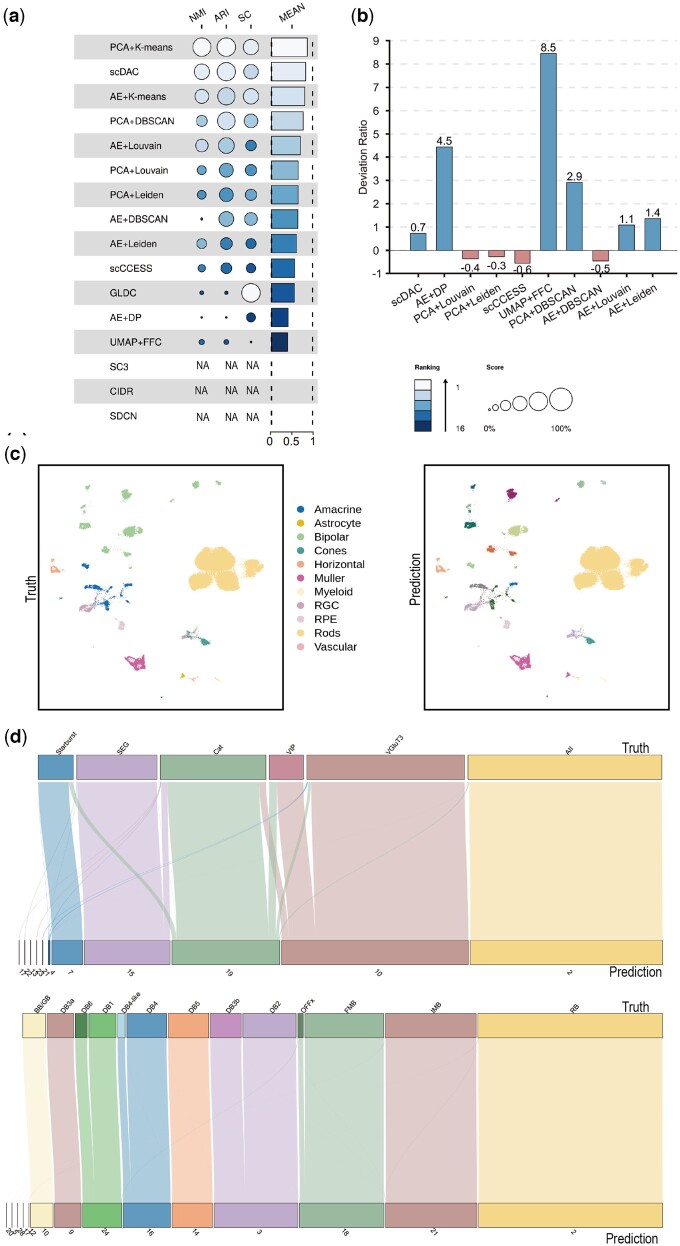
Comparisons of clustering performance between scDAC and the other 15 widely used methods on the Orozaco dataset. (a) NMI, ARI, SC, and mean scores of scDAC and the other methods. The 15 clustering methods are sorted in descending order of the mean scores. NA denotes that SC3, CIDR, and SDCN failed to produce clustering results on this dataset. (b) The bar plot of the Deviation Ratio of predicted labels by different methods. Shorter bars represent better results. (c) UMAP plots of the low-dimensional representations of scDAC. The left panel is the result marked with true labels, and the right one predicted labels. (d) The Sankey plots of scDAC shows the correspondence between the predicted labels and the ground truth on Amacrine (upper) and Bipolar (lower) cells. SC3, CIDR, and SDCN crashed and failed to produce results on this dataset. Therefore, the corresponding results are not depicted or marked as NA in the figure.

When we applied scDAC on a scRNA-seq dataset of human retina and retinal pigment epithelium (the Orozco dataset), we unexpectedly found that scDAC has lower NMI, ARI, SC, and the mean score than PCA+k-means, ranking in the second top among all the 15 methods (Fig. 4a and Supplementary Tables S3–S6). The Deviation Ratio of scDAC is higher than PCA+Louvain, PCA+Leiden, scCCESS, and AE+DBSCAN (Fig. 4b). The high positive deviation indicates remarkably more clusters than the ground truth. We investigated the reasons leading to more predicted clusters by scDAC than the true labels. The UMAP plots marked with the true label and the predicted label are shown in Fig. 4c. scDAC accurately clusters the cells from most cell types except Amacrine and Bipolar. scDAC subdivides the cells of the Amacrine and Bipolar into more clusters (circled by blue and green, respectively). Interestingly, the subclusters predicted by scDAC correspond quite well to the subtypes that have been verified to be *bona fide* subtypes of Amacrine and Bipolar cell type by experiments (Fig. 4d) (Orozco *et al.* 2020). When taking the fine-grained clusters of Amacrine and Bipolar into account, the indicators ARI and NMI between the true label and the predicted label are all >0.85, which are very high scores suggesting high similarity. These results show that scDAC can adaptively identify accurate subtypes with biological meanings.

### 3.4 Comparisons of running time and memory usage of scDAC and other methods on scRNA-seq datasets

**Figure 5.**
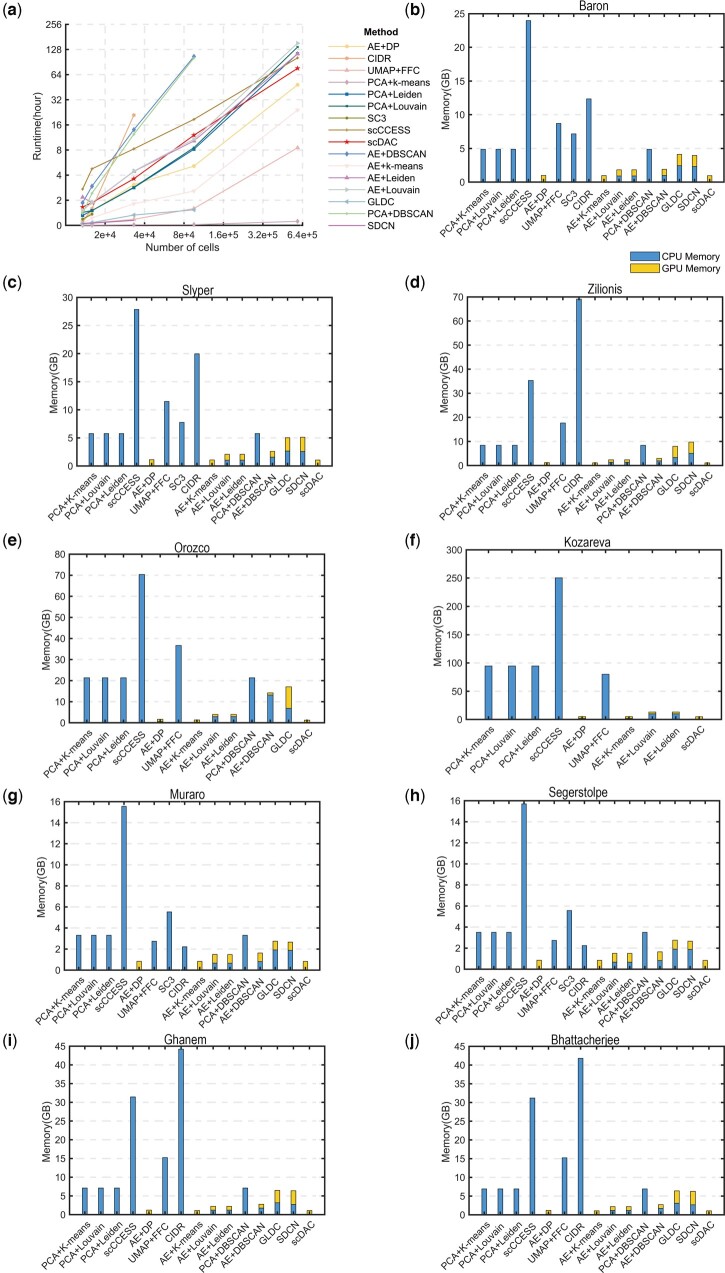
Comparisons of the runtime and memory costs of different methods. (a) Runtime of scDAC and the other methods. (b–j) GPU and CPU memory usage of scDAC and the other methods in Baron, Slyper, Zilionis, Orozco, Kozareva, Muraro, Segerstolpe, Ghanem, and Bhattacherjee datasets.

We evaluated the computing time and memory consumed by scDAC and the 15 other approaches on a Linux server with 72 CPU cores (two Intel Xeon Gold 6240 chips), 503 GB of RAM, and NVIDIA RTX 2080 Ti GPUs. As shown in Fig. 5a, the running time of scDAC is more than PCA+k-means, AE+kmeans, UMAP+FFC, and AE+DP when the cell numbers are less than 6.4e+5. However, as the cell numbers increase, the running time of scDAC increases linearly, and the running time of AE+kmeans, UMAP+FFC, and AE+DP increases exponentially. As shown in Fig. 5b–j, scDAC requires less memory than most methods and is equivalent to AE+kmeans and AE+DP. Notably, the clustering performance of scDAC is outstanding compared to these methods. Therefore, considering computing time, memory and performance, scDAC is a very advantageous method.

## 4 Discussion

Adaptive and accurate identification of clusters with biological meanings is urgently needed and is still quite challenging in large-scale scRNA-seq data analysis. To address the problem, we developed an adaptive clustering method scDAC for large-scale scRNA-seq data by coupling AE and DPMM and jointly optimizing these two modules. The results on subsampled and real scRNA-seq datasets show that scDAC can achieve adaptive clustering on large-scale scRNA-seq datasets from different sequencing platforms, species, organs, experimental conditions and scales and surpasses the other 15 widely used methods. Moreover, scDAC can accurately identify fine-grained clusters corresponding well to biological meanings.

Dimensionality reduction is an important step for high-dimensional scRNA-seq data. Normally, dimensionality reduction is not biased and has no information on cell clusters. If cluster information could be introduced into dimensionality reduction, the reduced dimensionality will maintain the information about clusters and will increase clustering performance. For this purpose, we employed AE to reduce dimensionalities and jointly optimized the parameters of AE and DPMM, i.e. the dimensionality reduction by AE is constrained by DPMM clustering. By adding this constraint, the AE and DPMM influence each other: the clustering is based on the low-dimensional representation, and the representation is affected by the distribution learned by the DPMM clustering module. Compared with the AE+DP method which separates AE and DPMM module, the coupling of AE and DPMM by scDAC improves the performance of clustering algorithms.

The coupling of dimensionality reduction and adaptive clustering can also be extended to clustering on single-cell sequencing data from multi-omics and multiple batches. With the rapid development of single-cell sequencing technologies, adaptive and accurate clustering methods for multi-omics and multiple batches will be urgently needed in the near future.

## Supplementary Material

btae198_Supplementary_Data

## Data Availability

The data underlying this article are available in the article and in its online supplementary material.
