# Supplementary material for: scDAC: deep adaptive clustering of single-cell transcriptomic data with coupled autoencoder and Dirichlet process mixture model
Source: Bioinformatics. 2024 Apr 11;40(4):btae198. doi: 10.1093/bioinformatics/btae198 (PMC11256937; doi:10.1093/bioinformatics/btae198)
Supplement: btae198_Supplementary_Data [file btae198_supplementary_data.pdf]

Supplementary Material for scDAC: deep adaptive clustering of single-cell transcriptomic data with coupled autoencoder and Dirichlet process mixture model

SIJING AN [1], JINHUI SHI [1], RUNYAN LIU [1], YAOWEN CHEN [1], JING WANG [1], SHUOFENG HU [1], XINYU XIA [1], GUOHUA DONG [1], XIAOCHEN BO [2,*], ZHEN HE [1,*] AND XIAOMIN YING [1,*]

1. SUPPLEMENTARY FIGURES

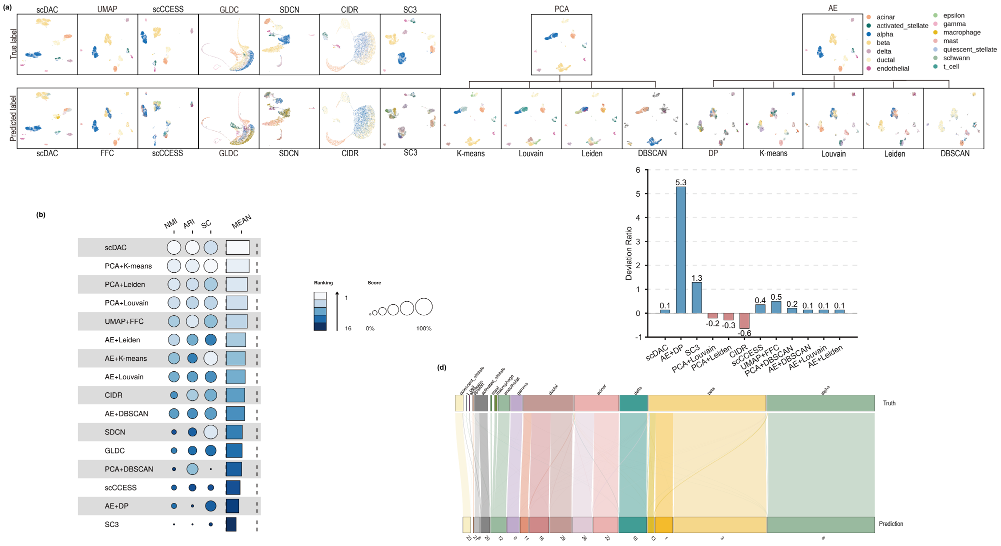

Fig. S1. Comparisons of clustering performance between scDAC and the other widely-used methods on the Baron dataset. (a) UMAP visualization of the low-dimensional representations of these methods. The upper panel is annotated with true labels and the lower panel is annotated with predicted labels. The first column is the UMAP plot of scDAC, and the other columns are the plots of other methods. (b) NMI, ARI, SC and mean scores of scDAC and the other methods. Each row represents a clustering method. The three columns of circles from left to right represent clustering indicators NMI, ARI and SC scores respectively. The rectangles on the right represent the mean scores of these three indicators. The size of the circles and rectangles correspond to the scores: the bigger one means the better performance. The darkness of color of the circles and rectangles correspond to the ranking: the lightest one means the top rank. The clustering methods are sorted according to the mean scores in descending order. (c) The bar plot of Deviation Ratios between the predicted labels by different methods and the true one. The x axis represents different methods, and the y axis represents the Deviation Ratio value. The blue bar denotes positive deviation and the red bar negative deviation. Shorter bars represent better results. AE+K-means, PCA+K-means, GLDC and SDCN were not involved in DR comparison since they require input of cell type number. (d) The Sankey plot of scDAC shows the correspondence between the predicted labels and the ground truth.

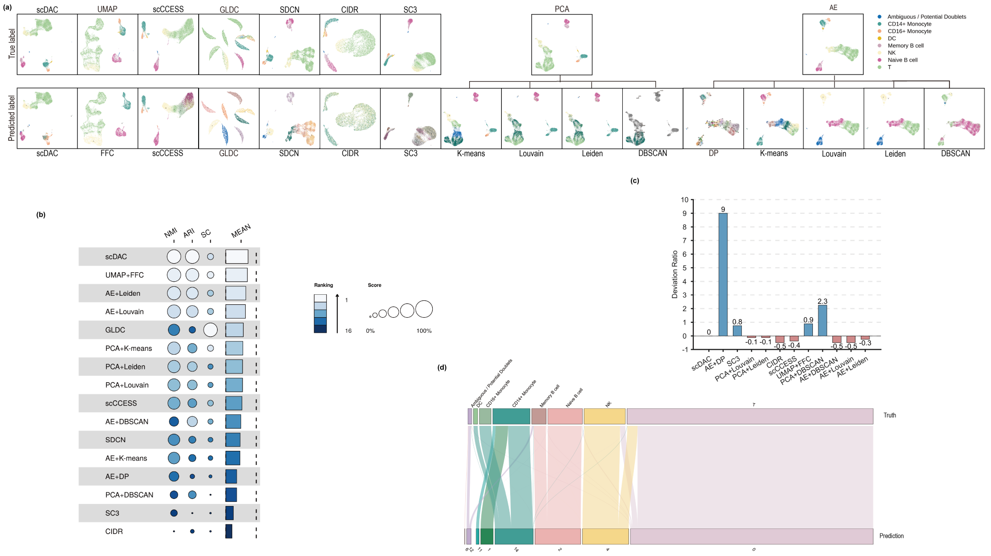

Fig. S2. Comparisons of clustering performance between scDAC and the other widely-used methods on the Slyper dataset. (a) UMAP visualization of the low-dimensional representations of these methods. The upper panel is annotated with true labels and the lower panel is annotated with predicted labels. The first column is the UMAP plot of scDAC, and the other columns are the plots of other methods. (b) NMI, ARI, SC and mean scores of scDAC and the other methods. Each row represents a clustering method. The three columns of circles from left to right represent clustering indicators NMI, ARI and SC scores respectively. The rectangles on the right represent the mean scores of these three indicators. The size of the circles and rectangles correspond to the scores: the bigger one means the better performance. The darkness of color of the circles and rectangles correspond to the ranking: the lightest one means the top rank. The clustering methods are sorted according to the mean scores in descending order. (c) The bar plot of Deviation Ratios between the predicted labels by different methods and the true one. The x axis represents different methods, and the y axis represents the Deviation Ratio value. The blue bar denotes positive deviation and the red bar negative deviation. Shorter bars represent better results. AE+K-means, PCA+K-means, GLDC and SDCN were not involved in DR comparison since they require input of cell type number. (d) The Sankey plot of scDAC shows the correspondence between the predicted labels and the ground truth.

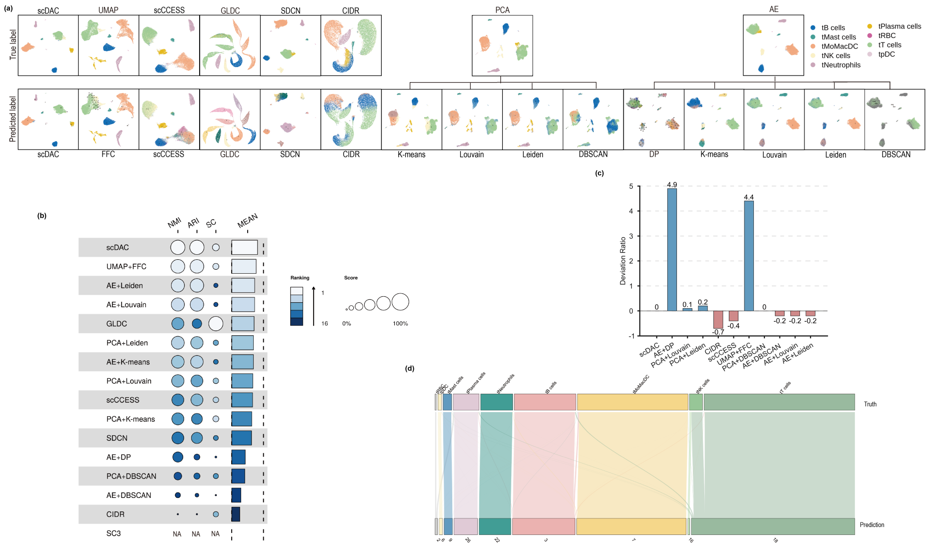

Fig. S3. Comparisons of clustering performance between scDAC and the other widely-used methods on the Zilionis dataset. (a) UMAP visualization of the low-dimensional representations of these methods. The upper panel is annotated with true labels and the lower panel is annotated with predicted labels. The first column is the UMAP plot of scDAC, and the other columns are the plots of other methods. (b) NMI, ARI, SC and mean scores of scDAC and the other methods. Each row represents a clustering method. The three columns of circles from left to right represent clustering indicators NMI, ARI and SC scores respectively. The rectangles on the right represent the mean scores of these three indicators. The size of the circles and rectangles correspond to the scores: the bigger one means the better performance. The darkness of color of the circles and rectangles correspond to the ranking: the lightest one means the top rank. The clustering methods are sorted according to the mean scores in descending order. NA denotes that the method failed to produce clustering results on this dataset. (c) The bar plot of Deviation Ratios between the predicted labels by different methods and the true one. The x axis represents different methods, and the y axis represents the Deviation Ratio value. The blue bar denotes positive deviation and the red bar negative deviation. Shorter bars represent better results. AE+K-means, PCA+K-means, GLDC and SDCN were not involved in DR comparison since they require input of cell type number (d) The Sankey plot of scDAC shows the correspondence between the predicted labels and the ground truth. SC3 crashed and failed to produce results on this dataset. Therefore, the corresponding results are not depicted or maked as NA in the figure.

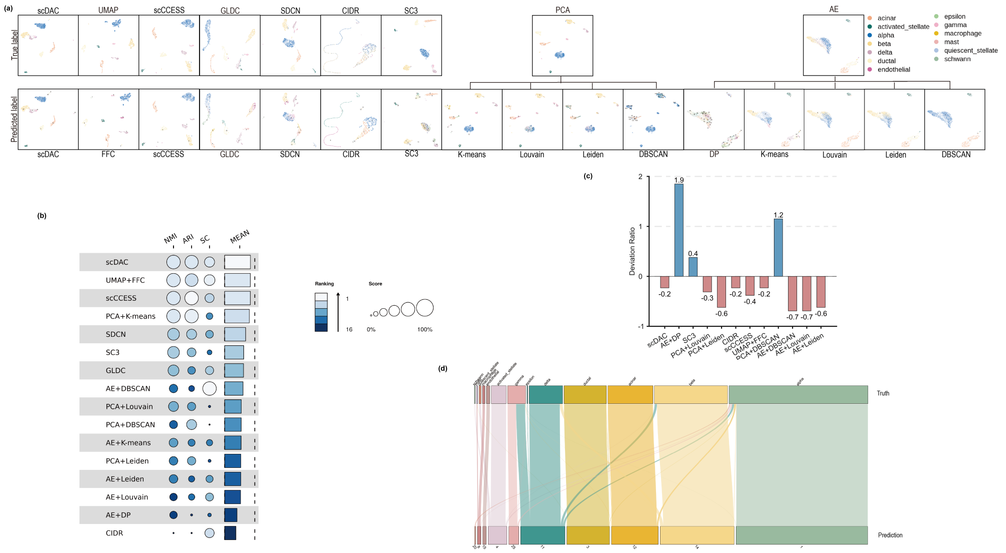

Fig. S4. Comparisons of clustering performance between scDAC and the other widely-used methods on the Muraro dataset. (a) UMAP visualization of the low-dimensional representations of these methods. The upper panel is annotated with true labels and the lower panel is annotated with predicted labels. The first column is the UMAP plot of scDAC, and the other columns are the plots of other methods. (b) NMI, ARI, SC and mean scores of scDAC and the other methods. Each row represents a clustering method. The three columns of circles from left to right represent clustering indicators NMI, ARI and SC scores respectively. The rectangles on the right represent the mean scores of these three indicators. The size of the circles and rectangles correspond to the scores: the bigger one means the better performance. The darkness of color of the circles and rectangles correspond to the ranking: the lightest one means the top rank. The clustering methods are sorted according to the mean scores in descending order. (c) The bar plot of Deviation Ratios between the predicted labels by different methods and the true one. The x axis represents different methods, and the y axis represents the Deviation Ratio value. The blue bar denotes positive deviation and the red bar negative deviation. Shorter bars represent better results. AE+K-means, PCA+K-means, GLDC and SDCN were not involved in DR comparison since they require input of cell type number. (d) The Sankey plot of scDAC shows the correspondence between the predicted labels and the ground truth.

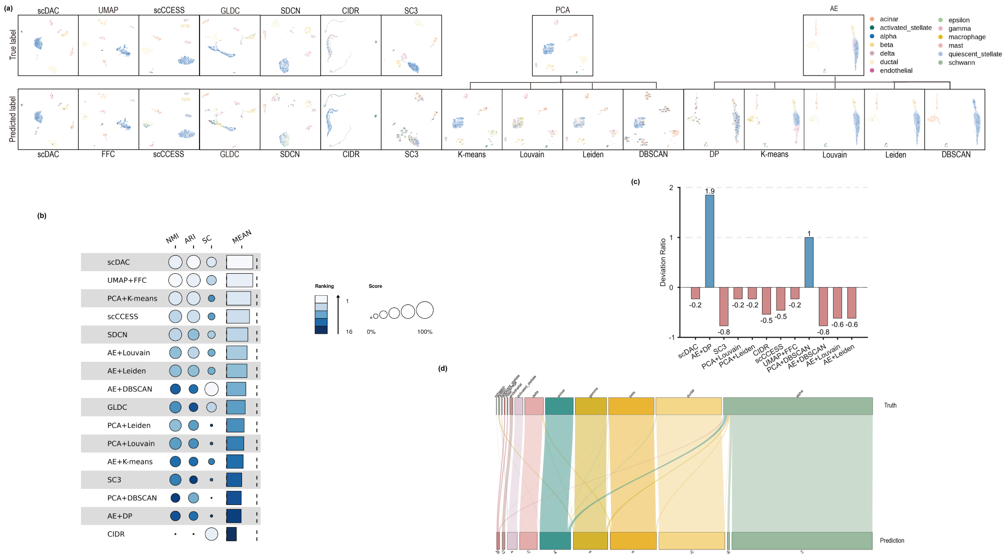

Fig. S5. Comparisons of clustering performance between scDAC and the other widely-used methods on the Segerstolpe dataset. (a) UMAP visualization of the low-dimensional representations of these methods. The upper panel is annotated with true labels and the lower panel is annotated with predicted labels. The first column is the UMAP plot of scDAC, and the other columns are the plots of other methods. (b) NMI, ARI, SC and mean scores of scDAC and the other methods. Each row represents a clustering method. The three columns of circles from left to right represent clustering indicators NMI, ARI and SC scores respectively. The rectangles on the right represent the mean scores of these three indicators. The size of the circles and rectangles correspond to the scores: the bigger one means the better performance. The darkness of color of the circles and rectangles correspond to the ranking: the lightest one means the top rank. The clustering methods are sorted according to the mean scores in descending order. (c) The bar plot of Deviation Ratios between the predicted labels by different methods and the true one. The x axis represents different methods, and the y axis represents the Deviation Ratio value. The blue bar denotes positive deviation and the red bar negative deviation. Shorter bars represent better results. AE+K-means, PCA+K-means, GLDC and SDCN were not involved in DR comparison since they require input of cell type number. (d) The Sankey plot of scDAC shows the correspondence between the predicted labels and the ground truth.

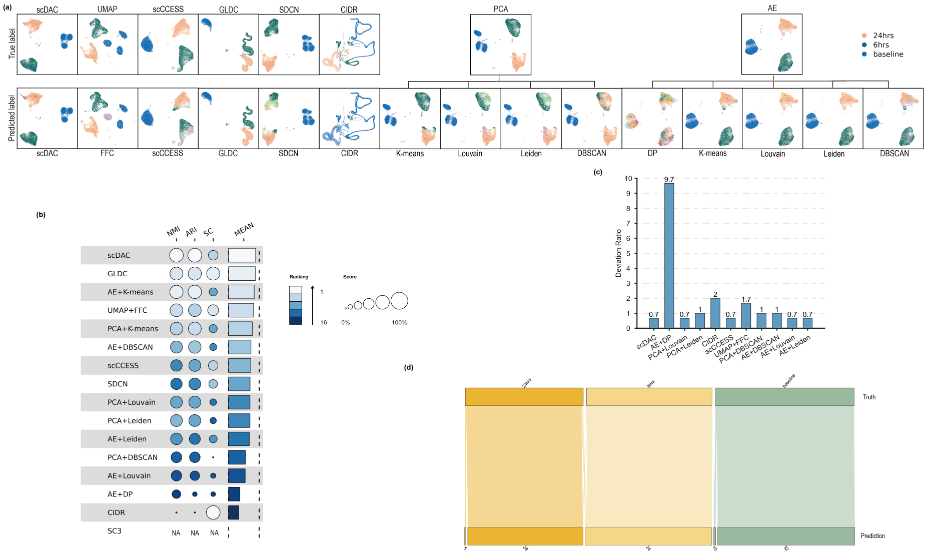

Fig. S6. Comparisons of clustering performance between scDAC and the other widely-used methods on the Ghanem dataset. (a) UMAP visualization of the low-dimensional representations of these methods. The upper panel is annotated with true labels and the lower panel is annotated with predicted labels. The first column is the UMAP plot of scDAC, and the other columns are the plots of other methods. (b) NMI, ARI, SC and mean scores of scDAC and the other methods. Each row represents a clustering method. The three columns of circles from left to right represent clustering indicators NMI, ARI and SC scores respectively. The rectangles on the right represent the mean scores of these three indicators. The size of the circles and rectangles correspond to the scores: the bigger one means the better performance. The darkness of color of the circles and rectangles correspond to the ranking: the lightest one means the top rank. The clustering methods are sorted according to the mean scores in descending order. (c) The bar plot of Deviation Ratios between the predicted labels by different methods and the true one. The x axis represents different methods, and the y axis represents the Deviation Ratio value. The blue bar denotes positive deviation and the red bar negative deviation. Shorter bars represent better results. AE+K-means, PCA+K-means, GLDC and SDCN were not involved in DR comparison since they require input of cell type number. (d) The Sankey plot of scDAC shows the correspondence between the predicted labels and the ground truth.

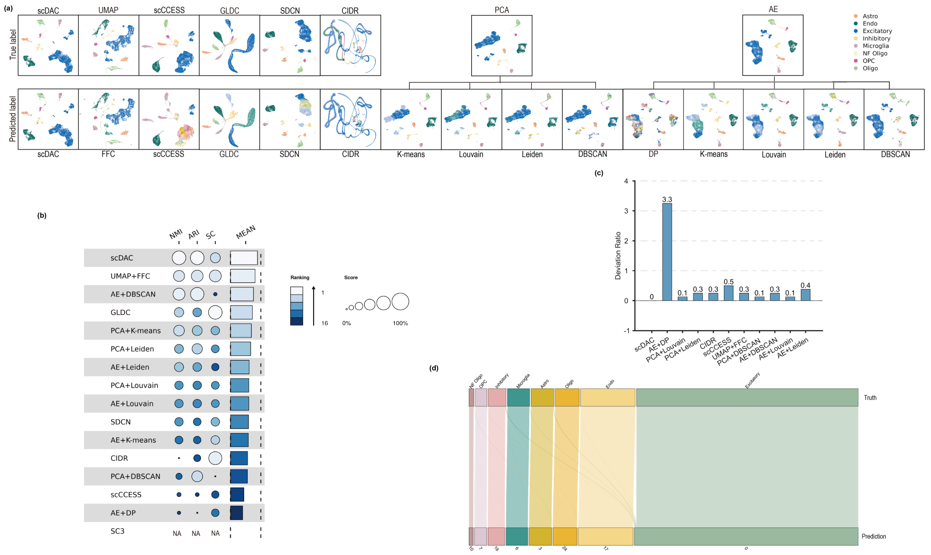

Fig. S7. Comparisons of clustering performance between scDAC and the other widely-used methods on the Bhattacherjee dataset. (a) UMAP visualization of the low-dimensional representations of these methods. The upper panel is annotated with true labels and the lower panel is annotated with predicted labels. The first column is the UMAP plot of scDAC, and the other columns are the plots of other methods. (b) NMI, ARI, SC and mean scores of scDAC and the other methods. Each row represents a clustering method. The three columns of circles from left to right represent clustering indicators NMI, ARI and SC scores respectively. The rectangles on the right represent the mean scores of these three indicators. The size of the circles and rectangles correspond to the scores: the bigger one means the better performance. The darkness of color of the circles and rectangles correspond to the ranking: the lightest one means the top rank. The clustering methods are sorted according to the mean scores in descending order. (c) The bar plot of Deviation Ratios between the predicted labels by different methods and the true one. The x axis represents different methods, and the y axis represents the Deviation Ratio value. The blue bar denotes positive deviation and the red bar negative deviation. Shorter bars represent better results. AE+K-means, PCA+K-means, GLDC and SDCN were not involved in DR comparison since they require input of cell type number. (d) The Sankey plot of scDAC shows the correspondence between the predicted labels and the ground truth.

2. SUPPLEMENTARY TABLES

Table S1. Description of the 10 scRNA-seq datasets used in the study

Dataset	Tissue	Size	Class	Protocol	Accession ID	Condition
Baron	Human pancreas	8569	14	10X Genomics	GSE84133	Healthy
Chen	Mouse brain	14207	15	Drop-seq	GSE87544	Healthy
Kozareva	Mouse cerebellum	611034	18	10X Genomics	SCP795	Healthy
Orozco	Human eye	100055	11	10X Genomics	GSE135133	Healthy
Slyper	Human blood	13316	8	10X Genomics	SCP345	Healthy
Zilionis	Human lung	34558	9	inDrop	GSE127465	Lung cancer
Muraro	Human pancreas	2122	13	CEL-seq2	GSE85241	Healthy
Segerstolpe	Human pancreas	2209	13	Smart-seq2	E-MTAB-5061	Type 2 diabetes
Ghanem	Human plasmacytoid	25511	3	10X Genomics	GSE189120	Influenza virus stimulation
Bhattacherjee	Mouse brain	24822	8	10X Genomics	GSE124952	Cocaine stimulation

Table S2. Cell types and cell numbers included in the subsampled datasets

No. Sequence	Cell Type	Cell Number
1	Ependy	401
2	Glu	858
3	IMO	151
4	Macro	156
5	Micro	699
6	MO	3516
7	OPC	1741
8	POPC	51
9	SCO	32
10	Tany	599

Table S3. NMI scores of scDAC and the other fifteen widely-used methods, NA denotes that the method failed to produce clustering result on the dataset.

Datasets	Baron	Kozareva	Orozco	Slyper	Zilionis	Bhattacherjee	Ghanem	Muraro	Segerstolpe
AE+DBSCAN	0.71	NA	0.54	0.42	0.28	0.88	0.76	0.57	0.57
AE+DP	0.57	0.44	0.54	0.45	0.57	0.52	0.45	0.52	0.54
AE+K-means	0.75	0.75	0.8	0.58	0.76	0.7	0.9	0.59	0.61
AE+Leiden	0.76	0.89	0.7	0.72	0.8	0.75	0.73	0.58	0.73
AE+Louvain	0.73	0.88	0.76	0.7	0.8	0.72	0.61	0.52	0.73
CIDR	0.62	NA	NA	0.07	0.13	0.45	0.06	0.24	0.06
GLDC	0.58	NA	0.58	0.56	0.72	0.76	0.86	0.72	0.69
PCA+DBSCAN	0.51	NA	0.73	0.33	0.42	0.63	0.64	0.55	0.47
PCA+K-means	0.84	0.78	0.9	0.65	0.71	0.82	0.81	0.9	0.88
PCA+Leiden	0.78	0.78	0.68	0.63	0.78	0.74	0.76	0.58	0.73
PCA+Louvain	0.78	0.78	0.68	0.63	0.74	0.72	0.75	0.66	0.72
SC3	0.46	NA	NA	0.27	NA	NA	NA	0.78	0.68
scCCESS	0.56	0.51	0.65	0.62	0.71	0.55	0.71	0.9	0.81
scDAC	0.84	0.96	0.83	0.75	0.9	0.96	0.95	0.9	0.9
SDCN	0.55	NA	NA	0.58	0.7	0.72	0.7	0.78	0.81
UMAP+FFC	0.76	0.53	0.6	0.74	0.86	0.84	0.84	0.9	0.91

Table S4. ARI scores of scDAC and the other fifteen widely-used methods, NA denotes that the method failed to produce clustering result on the dataset.

Datasets	Baron	Kozareva	Orozco	Slyper	Zilionis	Bhattacherjee	Ghanem	Muraro	Segerstolpe
AE+DBSCAN	0.52	NA	0.72	0.49	0.14	0.95	0.8	0.35	0.4
AE+DP	0.11	0.06	0.16	0.13	0.25	0.07	0.11	0.17	0.4
AE+K-means	0.48	0.47	0.88	0.27	0.74	0.4	0.93	0.42	0.4
AE+Leiden	0.59	0.99	0.56	0.64	0.83	0.49	0.63	0.37	0.62
AE+Louvain	0.56	0.99	0.78	0.63	0.83	0.47	0.46	0.38	0.63
CIDR	0.61	NA	NA	0.1	0.06	0.36	0.01	0.14	0.01
GLDC	0.42	NA	0.21	0.23	0.48	0.49	0.89	0.46	0.39
PCA+DBSCAN	0.6	NA	0.9	0.31	0.26	0.68	0.58	0.63	0.53
PCA+K-means	0.79	0.46	0.96	0.42	0.59	0.59	0.84	0.92	0.88
PCA+Leiden	0.68	0.48	0.67	0.45	0.71	0.63	0.73	0.51	0.51
PCA+Louvain	0.68	0.48	0.67	0.45	0.68	0.47	0.73	0.5	0.48
SC3	0.08	NA	NA	0.03	NA	NA	NA	0.54	0.31
scCCESS	0.32	0.42	0.51	0.38	0.68	0.17	0.73	0.93	0.8
scDAC	0.81	0.99	0.94	0.84	0.91	0.98	0.97	0.9	0.93
SDCN	0.4	NA	NA	0.27	0.61	0.41	0.71	0.63	0.62
UMAP+FFC	0.7	0.1	0.26	0.75	0.85	0.77	0.83	0.86	0.92

Table S5. SC scores of scDAC and the other fifteen widely-used methods, NA denotes that the method failed to produce clustering result on the dataset.

Datasets	Baron	Kozareva	Orozco	Slyper	Zilionis	Bhattacherjee	Ghanem	Muraro	Segerstolpe
AE+DBSCAN	0.58	NA	0.66	0.57	0.44	0.44	0.64	0.91	0.94
AE+DP	0.55	0.52	0.52	0.5	0.46	0.61	0.55	0.54	0.5
AE+K-means	0.61	0.58	0.76	0.55	0.55	0.66	0.68	0.63	0.63
AE+Leiden	0.56	0.83	0.56	0.61	0.53	0.6	0.67	0.66	0.67
AE+Louvain	0.57	0.83	0.58	0.61	0.53	0.62	0.57	0.69	0.67
CIDR	0.58	NA	NA	0.45	0.57	0.88	0.92	0.74	0.9
GLDC	0.55	NA	0.89	0.94	0.92	0.91	0.89	0.7	0.77
PCA+DBSCAN	0.39	NA	0.69	0.44	0.56	0.36	0.44	0.47	0.46
PCA+K-means	0.61	0.61	0.76	0.61	0.59	0.65	0.68	0.64	0.64
PCA+Leiden	0.6	0.56	0.6	0.57	0.57	0.62	0.61	0.52	0.5
PCA+Louvain	0.6	0.56	0.6	0.57	0.58	0.62	0.62	0.5	0.51
SC3	0.43	NA	NA	0.45	NA	NA	NA	0.58	0.51
scCCESS	0.47	0.52	0.54	0.59	0.58	0.6	0.74	0.73	0.66
scDAC	0.61	0.88	0.73	0.61	0.62	0.72	0.74	0.77	0.771
SDCN	0.61	NA	NA	0.56	0.55	0.65	0.7	0.68	0.68
UMAP+FFC	0.6	0.39	0.31	0.63	0.6	0.82	0.79	0.8	0.77

Table S6. Mean scores of NMI, ARI and SC by scDAC and the other fifteen widely-used methods, NA denotes that the method failed to produce clustering result on the dataset.

	Baron	Kozareva	Orozco	Slyper	Zilionis	Bhattacherjee	Ghanem	Muraro	Segerstolpe
AE+DBSCAN	0.60	NA	0.64	0.49	0.29	0.76	0.73	0.61	0.64
AE+DP	0.41	0.34	0.41	0.36	0.43	0.40	0.37	0.41	0.48
AE+K-means	0.62	0.60	0.81	0.47	0.68	0.59	0.84	0.55	0.55
AE+Leiden	0.64	0.90	0.61	0.66	0.72	0.61	0.68	0.54	0.67
AE+Louvain	0.62	0.90	0.71	0.65	0.72	0.60	0.55	0.53	0.68
CIDR	0.60	NA	NA	0.20	0.25	0.56	0.33	0.37	0.32
GLDC	0.52	NA	0.56	0.58	0.70	0.72	0.88	0.63	0.62
PCA+DBSCAN	0.50	NA	0.77	0.36	0.41	0.56	0.55	0.55	0.49
PCA+K-means	0.74	0.61	0.87	0.56	0.63	0.69	0.78	0.82	0.80
PCA+Leiden	0.69	0.61	0.65	0.55	0.69	0.66	0.70	0.54	0.58
PCA+Louvain	0.69	0.61	0.65	0.55	0.66	0.60	0.70	0.55	0.57
SC3	0.32	NA	NA	0.25	NA	NA	NA	0.63	0.50
scCCESS	0.45	0.48	0.56	0.53	0.66	0.44	0.73	0.85	0.76
scDAC	0.75	0.95	0.83	0.73	0.81	0.89	0.89	0.86	0.87
SDCN	0.52	NA	NA	0.47	0.62	0.59	0.70	0.70	0.70
UMAP+FFC	0.68	0.34	0.39	0.71	0.77	0.81	0.82	0.85	0.87
